# Racial and Ethnic Differences in Managed Care Enrollment Among US Children

**DOI:** 10.1001/jamanetworkopen.2021.4162

**Published:** 2021-04-02

**Authors:** Alon Peltz, Kristin Kan, Arvin Garg, Alexander Pomerantz, Lucy A. Bilaver, Matthew M. Davis

**Affiliations:** 1 Department of Population Medicine, Harvard Medical School, Boston, Massachusetts; 2 Department of Population Medicine, Harvard Pilgrim Health Care Institute, Boston, Massachusetts; 3 Department of Pediatrics, Northwestern University Feinberg School of Medicine, Chicago, Illinois; 4 Stanley Manne Children’s Research Institute, Ann & Robert H. Lurie Children’s Hospital of Chicago, Chicago, Illinois; 5 Department of Pediatrics, University of Massachusetts Medical School, Worcester; 6 Harvard Kennedy School and Harvard Medical School, Boston, Massachusetts

## Abstract

This survey study uses data from the 2018 Medical Expenditure Panel Survey Household Component to compare rates of health maintenance organization (HMO) enrollment, by race and ethnicity, for children with commercial and public coverage.

## Introduction

Racial and ethnic inequities exist in child health care outcomes.^1^ In the United States, medical insurance coverage is an important determinant of timely and affordable health care services.^2^ One common form of coverage—the health maintenance organization (HMO)—is intended as a lower-cost, more efficient, and better-organized delivery model, focused on managing care within a closed network of hospitals and clinicians who are often paid a fixed fee to administer services. Despite their popularity, the evidence regarding the efficacy of HMOs is mixed when considering clinical outcomes.^3^ Other evidence raises concerns about high turnover among in-network health care professionals,^4^ restrictive networks, and reduced access to specialty care.^5^ Because coverage for children is contingent on parental income and employment, existing economic inequalities raise concerns about unexplored racial and ethnic differences in HMO enrollment. We used national survey data to compare rates of HMO enrollment, by race and ethnicity, for children with commercial and public coverage.

## Methods

The 2018 Medical Expenditure Panel Survey Household Component (MEPS-HC) public use file, a nationally representative survey of US households, was used to identify children (from birth to 17 years) with public or commercial coverage for this survey study. The MEPS-HC data were collected by computer-assisted personal interviews using probability-based sampling methods consistent with the American Association for Public Opinion Research (AAPOR) reporting guideline. We defined HMO enrollment during the year using self-reported information. Prior work shows that MEPS respondents provide accurate information regarding HMO enrollment.^6^ We compared rates of HMO enrollment for 3 mutually exclusive racial/ethnic groups (non-Hispanic White, non-Hispanic Black, and Hispanic individuals) when the response exceeded the recommended sample size thresholds for reliable reporting. Data for race/ethnicity were self-reported, with imputation for missing or incomplete responses (14% for race; 10% for ethnicity) by MEPS based on immediate family members living in the household. To model HMO enrollment, we constructed logistic regression models with robust variance estimators for each coverage group, conditional on age, special health care needs, US Census region, household size, and household income (by percentage of federal poverty level). For commercial coverage, we included 2 additional variables: the purchase of medical insurance coverage on an exchange and whether the respondent’s employer offered more than 1 insurance plan. Statistical analyses were performed with Stata version 14 (StataCorp). Weighting, stratification, and clustering estimators were applied to account for the complex survey design, with significance set at *P* < .05 using a 2-sided test. The study was designated as non–human participant research by the Harvard Pilgrim Health Care Institutional Review Board.

## Results

The cohort included 5543 HMO-enrolled and non–HMO-enrolled children, representing 57.4 million US children. When weighted to represent the national population, 3143 children (56.7%) were non-Hispanic White individuals, 1569 (28.3%) were Hispanic individuals, and 831 (15.0%) were non-Hispanic Black individuals. Approximately two-thirds of the children (3498 [63.1%]) had commercial coverage, and 2162 children (39%) were enrolled in an HMO. Table 1 reports the percentage of HMO enrollment across coverage groups. In unadjusted analysis, Hispanic children (overall, 52.5%; public coverage, 59.4%; and commercial coverage, 42.4%) were most often enrolled in HMOs, followed by non-Hispanic Black children (overall, 39.0%; public coverage, 35.4%; and commercial coverage, 43.2%), and non-Hispanic White children (overall, 31.4%; public coverage, 36.3%; and commercial coverage, 30.1%). Enrollment in HMOs also differed based on US Census region and income. When adjusted for sociodemographic characteristics, Hispanic children with public coverage (adjusted odds ratio [AOR], 2.6; 95% CI, 1.9-3.6); *P* < .001), non-Hispanic Black children with commercial coverage (AOR, 1.9; 95% CI, 1.2-3.1; *P* = .004), and Hispanic children with commercial coverage (AOR, 1.7; 95% CI, 1.2-2.5; *P* = .01) were more likely to be enrolled in HMOs than non-Hispanic White children (Table 2).

**Table 1.** HMO Enrollment for US Children in 2018

Characteristic	HMO enrollment^a^					
	Any coverage^b^		Public coverage^c^		Commercial coverage^c^	
	No. (%)	*P* value^d^	No. (%)	*P* value^d^	No. (%)	*P* value^d^
Race/ethnicity						
Hispanic	1116 (52.5)	<.001	814 (59.4)	<.001	302 (42.4)	.001
Non-Hispanic Black	378 (39.0)		222 (35.4)		156 (43.2)	
Non-Hispanic White	925 (31.4)		304 (36.3)		621 (30.1)	
Age, y						
≤5	737 (38.3)	.40	419 (47.3)	.70	318 (33.1)	.42
6-11	781 (36.9)		454 (45.2)		327 (31.6)	
12-17	835 (40.2)		437 (47.4)		398 (36.3)	
US Census region						
Northeast	336 (42.4)	<.001	161 (56.6)	.01	175 (36.8)	.04
Midwest	508 (37.8)		240 (44.7)		268 (34.6)	
South	851 (32.0)		494 (38.1)		357 (28.1)	
West	724 (46.5)		445 (55.6)		279 (39.7)	
Family size, No. of members						
1-3	498 (39.2)	.18	265 (39.9)	.08	233 (38.8)	.34
4-5	1356 (37.0)		690 (47.1)		666 (32.2)	
≥6	565 (42.7)		385 (52.2)		180 (33.8)	
Special health care needs^e^	389 (38.6)	.32	214 (47.4)	.24	175 (35.7)	.77
Household income, % of FPL						
<150	1068 (45.4)	<.001	920 (45.4)	.48	148 (45.5)	.04
150-300	652 (39.4)		349 (49.0)		303 (32.3)	
300-400	258 (39.2)		NA^f^		213 (37.5)	
>400	441 (31.4)		NA^f^		415 (31.2)	

Abbreviations: FPL, federal poverty level; HMO, health maintenance organization; NA, not applicable.

^a^ The table presents only the weighted percentage of HMO enrollment; percentages of non-HMO enrollees are not shown. As such, the percentage totals within each demographic group do not equal 100%.

^b^ Any coverage denotes either public or commercial coverage.

^c^ Public coverage was defined as only Medicaid, Children’s Health Insurance Program, or both during the year; commercial coverage was defined as any private health insurance coverage during the year.

^d^ Statistical significance was set at *P* < .05.

^e^ Special health care needs were defined using the Children with Special Health Care Needs Screener based on the Maternal and Child Health Bureau definition.

^f^ Data were omitted owing to small sample sizes below the minimum reportable level.

**Table 2.** Adjusted Odds of HMO Enrollment in 2018 for US Children by Race and Ethnicity^a^

Race/ethnicity	Public coverage		Commercial coverage	
	AOR (95% CI)	*P* value^b^	AOR (95% CI)	*P* value^b^
Hispanic	2.6 (1.9-3.6)	<.001	1.7 (1.2-2.5)	.01
Non-Hispanic Black	1.1 (0.7-1.7)	.61	1.9 (1.2-3.1)	.004
Non-Hispanic White	1 [Reference]	NA	1 [Reference]	NA

Abbreviations: AOR, adjusted odds ratio; HMO, health maintenance organization; NA, not applicable.

^a^ To estimate racial and ethnic differences in HMO enrollment, we constructed weighted logistic regression models with robust estimators of variance, using separate models for each coverage cohort, conditional on the covariates listed in Table 1. For commercial coverage, we included 2 additional variables: purchase of medical insurance coverage on an exchange, and whether the respondent’s employer offers more than 1 insurance plan.

^b^ Statistical significance was set at *P* < .05.

## Discussion

Enrollment in HMOs is common among US children with both public and commercial medical insurance coverage. At the national level, non-Hispanic Black and Hispanic children are enrolled in HMOs at higher proportions than White children, although rates vary by coverage type. Differences in Medicaid policies regarding mandatory HMO enrollment and employer-sponsored coverage offerings in communities with racial and ethnic minority residents may contribute to our findings. Limitations of this study include the reliance on self-reported data for HMO enrollment and the inability to account for state identifiers that were absent from this data set. Our results call for future inquiry to examine the degree to which the observed national imbalance in HMO enrollment results from state-specific policies, family preferences, or coverage affordability. Absent the unequivocal benefit of HMOs, the observed national differences in enrollment raise concerns for structural racism with regard to children’s coverage, with potential implications for access to care.

## References

[1] Agency for Healthcare Research and Quality. 2019 National Healthcare Quality and Disparities Report. Agency for Healthcare Research and Quality. December 2020. Updated February 2021. Accessed February 24, 2021. https://www.ahrq.gov/research/findings/nhqrdr/nhqdr19/index.html

[2] Kreider AR, French B, Aysola J, Saloner B, Noonan KG, Rubin DM. Quality of health insurance coverage and access to care for children in low-income families. JAMA Pediatr. 2016;170(1):43-51. doi:10.1001/jamapediatrics.2015.3028 26569497 PMC8011294

[3] Gilchrist-Scott DH, Feinstein JA, Agrawal R. Medicaid managed care structures and care coordination. Pediatrics. 2017;140(3):e20163820. doi:10.1542/peds.2016-3820 28838950

[4] Ndumele CD, Staiger B, Ross JS, Schlesinger MJ. Network optimization and the continuity of physicians in Medicaid managed care. Health Aff (Millwood). 2018;37(6):929-935. doi:10.1377/hlthaff.2017.1410 29863934

[5] Herring B, Adams EK. Using HMOs to serve the Medicaid population: what are the effects on utilization and does the type of HMO matter? Health Econ. 2011;20(4):446-460. doi:10.1002/hec.1602 21394815

[6] Zuvekas S, Olin G. Accuracy of household reports of Medicare managed care enrollment in the MEPS. Agency for Healthcare Research and Quality. Working Paper No. 08012. December 2008. Accessed December 17, 2020. https://www.meps.ahrq.gov/data_files/publications/workingpapers/wp_08012.pdf

